# The impact of the affordable care act on perinatal mood and anxiety disorder diagnosis and treatment rates among Michigan Medicaid enrollees 2012–2018

**DOI:** 10.1186/s12913-023-10539-y

**Published:** 2024-01-30

**Authors:** Stephanie V. Hall, Kara Zivin, Gretchen A. Piatt, Addie Weaver, Anca Tilea, Xiaosong Zhang, Cheryl A. Moyer

**Affiliations:** 1 Department of Psychiatry, University of Michigan, 2800 Plymouth Road, Ann Arbor, MI 48109 USA; 2 Department of Learning Health Sciences, University of Michigan, 1111 E Catherine St, Ann Arbor, MI 48109 USA; 3 Department of Obstetrics and Gynecology, University of Michigan, 1500 E Medical Center Dr, Ann Arbor, MI 48109 USA; 4 School of Social Work, University of Michigan, 1080 S University Ave, Ann Arbor, MI 48109 USA

**Keywords:** Maternal mental health, Health policy, Depression, Anxiety, Perinatal health

## Abstract

**Background:**

Perinatal Mood and Anxiety Disorders (PMADs) affect one in five birthing individuals and represent a leading cause of maternal mortality. While these disorders are associated with a variety of poor outcomes and generate significant societal burden, underdiagnosis and undertreatment remain significant barriers to improved outcomes. We aimed to quantify whether the Patient Protection Affordable Care Act (ACA) improved PMAD diagnosis and treatment rates among Michigan Medicaid enrollees.

**Methods:**

We applied an interrupted time series framework to administrative Michigan Medicaid claims data to determine if PMAD monthly diagnosis or treatment rates changed after ACA implementation for births 2012 through 2018. We evaluated three treatment types, including psychotherapy, prescription medication, and either psychotherapy or prescription medication. Participants included the 170,690 Medicaid enrollees who had at least one live birth between 2012 and 2018, with continuous enrollment from 9 months before birth through 3 months postpartum.

**Results:**

ACA implementation was associated with a statistically significant 0.76% point increase in PMAD diagnosis rates (95% CI: 0.01 to 1.52). However, there were no statistically significant changes in treatment rates among enrollees with a PMAD diagnosis.

**Conclusion:**

The ACA may have improved PMAD detection and documentation in clinical settings. While a higher rate of PMAD cases were identified after ACA Implementation, Post-ACA cases were treated at similar rates as Pre-ACA cases.

**Supplementary Information:**

The online version contains supplementary material available at 10.1186/s12913-023-10539-y.

## Background

The Patient Protection and Affordable Care Act (ACA) of 2010 greatly expanded health insurance coverage and enhanced clinical service for millions of people in the United States (US). The ACA prohibited private insurance companies from denying coverage due to preexisting conditions, required insurance companies to cover dependents through 26 years of age, and gave states the option to expand Medicaid coverage for individuals living below 133% of the federal poverty level (FPL) [1–4]. The ACA also required insurance plans to cover mental health services at the same rate as physical health services, supported cross-disciplinary medical training for providers [5], and required coverage for preventive services, including mental health screening [6].

Perinatal Mood and Anxiety Disorders (PMADs) are a highly prevalent and burdensome pregnancy complication. Affecting one in five pregnant and postpartum people, PMADs are associated with preeclampsia, preterm birth, low birthweight, and maternal mortality [7–10]. Clinical detection and management represent key tools for PMAD case management; however, many cases remain undiagnosed and untreated [11]. The underlying reasons for underdiagnosis and undertreatment include barriers to accessing care, inadequate provider training, and lack of coordination between specialty care providers (e.g., psychiatry, obstetrics, pediatrics) [12–15]. Pregnant and postpartum people enrolled in Medicaid may experience additional barriers such as lower access to care and stigma [12–15].

The ACA increased Medicaid enrollment for reproductive-aged people [16, 17] and birthing people [3, 18–20] and improved continuity of care for birthing people [21]. The ACA is also associated with increased use of preconception services, adequate prenatal care, and timely prenatal care [18, 22–24]. Improved perinatal health care may have provided additional opportunities for PMAD screening, diagnosis, and treatment of maternal mental health conditions. Improved Medicaid access may have reduced pre-pregnancy and postpartum depressive symptoms [25, 26].

This study assessed whether monthly PMAD diagnosis and treatment rates changed after ACA implementation. We hypothesized that PMAD diagnosis and treatment rates would increase after ACA implementation due to improved access and incentivization for diagnosis and treatment. This study helps determine whether policy initiatives such as the ACA can improve clinical diagnosis and treatment rates for birthing people experiencing PMAD.

## Materials and methods

### Study design

This study used a retrospective, quasi-experimental interrupted time series (ITS) analysis of Michigan Medicaid claims to compare PMAD diagnosis and treatment rates before and after ACA implementation.

We conducted analysis at the month-level by grouping all births into 84 calendar months (January 2012–December 2018) by infant birth date. Michigan implemented the ACA on April 1, 2014, though we assigned ACA status using the ACA implementation date plus nine months (i.e., January 1, 2015) to ensure each Post-ACA birth was fully exposed to ACA throughout pregnancy. We assigned births that occurred before January 1, 2015 to the Pre-ACA and births that occurred on or after January 1, 2015 to the Post-ACA period. Our analysis included 36 Pre-ACA months (infant birth month: January 2012–December 2014) and 48 Post-ACA months (infant birth month: January 2015–December 2018).

### Participants

Participants included all Michigan Medicaid enrollees who had a live birth during the study period and had continuous enrollment from nine months before birth through three months postpartum, as Michigan Medicaid covers births through the calendar month of 60 days after birth. The continuous Medicaid enrollment criterion ensures that participants’ perinatal health care interactions were captured in the Medicaid claims dataset.

For Michigan Medicaid enrollees with more than one birth during the study period, we randomly selected one birth using the R function n_sample() and excluded all other births for that participant. That is, each included participant represents one birth. Limiting data to one birth per participant eliminated potential bias from repeated measures of the same participant.

The primary analysis on diagnosis rates included all study participants. The secondary analysis on treatment rates included only study participants with a PMAD diagnosis.

### Data collection

The Michigan Department of Health and Human Services (MDHHS) manages the Michigan Medicaid claims data warehouse. MDHHS provided our study team with a de-identified analytic dataset of processed claims from one year before birth through one year after birth for all Medicaid-insured births from 2012 to 2018.

The Institutional Review Boards of the University of Michigan (HUM00148854) and MDHHS (201811-10-EA) approved the study protocol and waived the need for informed consent because of the retrospective nature of the study. All methods were carried out in accordance with relevant guidelines and regulations.

#### Primary outcome

The primary outcome of interest was the monthly PMAD diagnosis rate. We used infant birth month as an index date, but the PMAD diagnosis could occur any time during the perinatal period of nine months before birth through three months after birth. We identified PMAD diagnoses using the International Classification of Diseases, Ninth Revision and Tenth Revision (ICD-9/10) codes listed in Appendix A.

#### Secondary outcome

The secondary outcome of interest was the monthly PMAD treatment rate. We examined three treatment categories: (1) psychotherapy, (2) prescription medication, and (3) psychotherapy or prescription medication. Again, we used infant birth month as an index date, but treatment could occur at any point during the perinatal period of nine month before through three months after birth.

We defined psychotherapy utilization as any number of psychotherapy sessions during the perinatal period. We identified psychotherapy treatment using a set of Current Procedural Terminology (CPT) codes based on the Healthcare Utilization Project (HCUP) [27] and Healthcare Effectiveness Data and Information Set (HEDIS) [28] and shown in Appendix B.

We defined prescription medication utilization as filling any antidepressants, anxiolytics, and mood stabilizing medications during the perinatal period. We identified these prescription medications using the Hierarchical Ingredient Code (HIC) numbers shown in Appendix C. Medicaid prescription claims indicate a prescription fill; however, we cannot know whether the patient took the medication as prescribed.

The final treatment category is “Any Treatment,” which included either psychotherapy or prescription medication utilization.

#### Independent variable of interest

The independent variable of interest was ACA implementation. This ITS analysis measures ACA implementation in three variables: (1) Time, (2) ACA Implementation, and (3) Time Since ACA Implementation. We coded Time by chronologically numbering the 84 study months beginning with January 2012 and concluding with December 2018. The Time variable represents temporal trends in diagnosis/treatment rate before ACA implementation.

We coded ACA Implementation by dichotomizing all enrollees and study months as Pre-ACA or Post-ACA. We assigned study months January 2012–December 2014 a “0” to indicate Pre-ACA and study months January 2015–December 2018 a “1” to indicate Post-ACA. The dichotomous ACA Implementation variable represents the change in Pre-ACA monthly rates versus Post-ACA monthly rates.

We coded Time Since Implementation by numbering all Pre-ACA study months as “0” and numbering Post-ACA study months chronologically from 1 (January 2015) through 48 (December 2018). The Time Since Implementation variable represents the change in temporal trend from Pre-ACA to Post-ACA.

#### Covariates

In adjusted analyses, we controlled for monthly participant demographic characteristics, including age, race/ethnicity, and comorbidities. We dichotomized each covariate and controlled for the percent of enrollees with that characteristic for each month.

We dichotomized the age cutoff at 26 years because the ACA included a provision that dependents can remain on their parent’s insurance plan through the year of their 26th birthday. The claims data recorded race/ethnicity as American Indian/Alaska Native, Asian, Black, Hispanic/Pacific Islander, Other/Unspecified, Unknown, and White. We categorized race/ethnicity as “white” and “non-white” due to racial disparities in maternal mental health care [12–15]. We measured comorbidities with the Obstetric Comorbidity Index (OBCMI). The OBCMI generates a prenatal risk score comprised of prenatal comorbidities such as gestational diabetes, autoimmune disease, and hypertension. The OBCMI has a strong correlation with severe maternal morbidity (SMM) [29]. We dichotomized OBCMI score cutoff of > 2 [29].

### Data analysis

We conducted data analysis at the month level using monthly rates for each outcome and covariate. We calculated monthly diagnosis rates, treatment rates, and enrollment characteristics by grouping births by infant birth month, counting the number of participants who had a birth in that month with that characteristic, and dividing by the total number of participants who had a birth in that month.

The analysis included 84 months in the study period of January 1, 2012–December 31, 2018, making the analytic sample *N* = 84.

We assessed the relationship between ACA implementation and monthly PMAD diagnosis and treatment rates using simple linear regression via the R function lm(). Unadjusted ITS models included three ACA predictors: Time, ACA Implementation, and Time Since ACA Implementation. Adjusted ITS models included the same three predictors while also controlling for the monthly percent of enrollees ≥ 26 years of age, percent of non-white enrollees, and percent of enrollees with an OBCMI score > 2.

We generated monthly predicted PMAD diagnosis and treatment rates for unadjusted and adjusted models using the R function predict().

We generated counterfactual monthly predicted PMAD diagnosis and treatment rates by using the R function predict() on a mock dataset in which the Time variable used numbered months 1–84, but the dichotomized ACA Implementation variable and the Time Since ACA Implementation variable remained at zero for the entire study period. This mock dataset essentially extends the Pre-ACA PMAD diagnosis and treatment trends through the Post-ACA time period and serves as an expected comparator.

We conducted cohort selection in SAS Software, version 9 [30] and analysis in R, version 4.0.3 [31].

## Results

### Study cohort characteristics

In Michigan, 332,062 Medicaid-insured births occurred between January 1, 2012, and December 31, 2018. Just under two-thirds of enrollees (*n* = 208,493) had continuous enrollment from nine months before birth through three months postpartum. After randomly selecting one birth per enrollee, the primary study cohort included 170,690 enrollees. The secondary study cohort of only those with PMAD diagnosis included 26,551 enrollees (15.6% of primary cohort). We grouped enrollees into 84 study months (i.e., January 2012–December 2018) based on infant birth date. The study cohort diagram appears in Appendix D.

On average, 2,032 participants had a birth per study month. Diagnosis and treatment rates were higher in the Post-ACA period compared to the Pre-ACA period (*p* < 0.001 for both). Participants were more likely to be ≥ 26 years old, non-white, and have an OBCMI score > 2 in the Post-ACA period compared to the Pre-ACA period (*p* < 0.001 for all). Study participant characteristics appear in Table 1.

**Table 1:** Study cohort characteristics by affordable care act implementation timepoints (*n* = 170,690)^a^

	Pre-ACA	Post-ACA	Overall
	1/1/12–12/31/14	1/1/15 − 12/31/18	1/1/12–12/31/18
	(n = 50,964)	(n = 119,726)	(n = 170,690)
**Variable**	No. (%)	No. (%)	No. (%)
**Perinatal Mood and Anxiety Disorder (PMAD) Diagnosis Rate**			
PMAD Diagnosis	6443 (12.6%)	20,108 (16.8%)	26,551 (15.6%)
No PMAD Diagnosis	44,521 (87.4%)	99,618 (83.2%)	144,139 (84.4%)
**Any Treatment Rate**			
Treatment	11,077 (21.7%)	30,490 (25.5%)	41,567 (24.4%)
No Treatment	39,887 (78.3%)	89,236 (74.5%)	129,123 (75.6%)
**Prescription Medication Rate**			
Prescription Medication	9542 (18.7%)	25,574 (21.4%)	35,116 (20.6%)
No Prescription Medication	41,422 (81.3%)	94,152 (78.6%)	135,574 (79.4%)
**Psychotherapy Rate**			
Psychotherapy	3347 (6.6%)	10,773 (9.0%)	14,120 (8.3%)
No Psychotherapy	47,617 (93.4%)	108,953 (91.0%)	156,570 (91.7%)
**Age at delivery (years)**			
< 26 years	25,107 (49.3%)	52,573 (43.9%)	77,680 (45.5%)
≥ 26 years	25,857 (50.7%)	67,153 (56.1%)	93,010 (54.5%)
**Race/ethnicity**			
White	32,037 (62.9%)	71,718 (59.9%)	103,755 (60.8%)
Non-white/Unknown	18,927 (37.1%)	48,008 (40.1%)	66,935 (39.2%)
**OBCMI Score**			
OBCMI score ≤ 2	45,083 (88.5%)	98,933 (82.6%)	144,016 (84.4%)
OBCMI score > 2	5881 (11.5%)	20,793 (17.4%)	26,674 (15.6%)

^a^All Pre-ACA versus Post-ACA differences are significant at *p* < 0.001

### Diagnosis rates

**Fig. 1:**
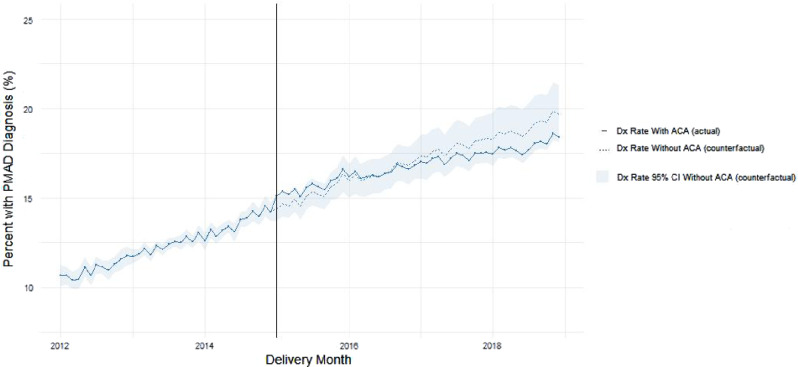
Adjusted monthly perinatal mood and anxiety disorder (PMAD) diagnosis rates among birthing Medicaid enrollees 2012–2018^a,b^. ^a^Adjusted for: time, ACA implementation, time since ACA implementation, percent of enrollees ≥ 26, percent of non-white enrollees, and percent of enrollees with OBCMI score > 2. ^b^Light blue shading and dashed line indicate model with ACA implementation set to 0; this represents a counterfactual projection of rates had the ACA not been implemented

The adjusted monthly PMAD diagnosis rates appear in Fig. 1. The rates indicate the percent of birthing enrollees diagnosed with a PMAD at some point during the perinatal period plotted by infant birth month. Models adjust monthly PMAD diagnosis rates for the three key independent variables of Time, ACA Implementation, and Time Since ACA Implementation as well as the percent of enrollees ≥ 26 years of age, percent of non-white enrollees, and percent of enrollees with an OBCMI scores > 2.

### Study cohort characteristics: enrollees with PMAD diagnoses only

The secondary study cohort, restricted to only participants with PMAD diagnoses, included 26,551 participants. The percent of enrollees that used either psychotherapy or prescription medication did not vary significantly from the Pre-ACA period to the Post-ACA period. However, prescription medication use decreased from 70.7% in the Pre-ACA period to 68.9% in the Post-ACA period (*p* = 0.002), and the psychotherapy use increased from 31.9% in the Pre-ACA period to 34.5% in the Post-ACA period (*p* = 0.001). Enrollees with a PMAD diagnosis were more likely to be ≥ 26 years of age, non-white, and have an OBCMI score > 2 in the Post-ACA period compared to the Pre-ACA period (*p* < 0.001 for all). The secondary study cohort characteristics appear in Table 2.

**Table 2:** Perinatal mood and anxiety disorder diagnosis study cohort characteristics by affordable care act implementation timepoint (*n* = 26,551)^a^

	Pre-ACA	Post-ACA	Overall	
	1/1/12–12/31/14	1/1/15 − 12/31/18	1/1/12–12/31/18	
	(n = 6443)	(n = 20,108)	(n = 26,551)	
**Variable**	No. (%)	No. (%)	No. (%)	P-value
**Any Treatment Rate**				
Treatment	5240 (81.3%)	16,175 (80.4%)	21,415 (80.7%)	0.291
No Treatment	1203 (18.7%)	3933 (19.6%)	5136 (19.3%)	
**Prescription Medication Rate**				
Prescription Medication	4555 (70.7%)	13,750 (68.4%)	18,305 (68.9%)	0.002**
No Prescription Medication	1888 (29.3%)	6358 (31.6%)	8246 (31.1%)	
**Psychotherapy Rate**				
Psychotherapy	2058 (31.9%)	7091 (35.3%)	9149 (34.5%)	< 0.001***
No Psychotherapy	4385 (68.1%)	13,017 (64.7%)	17,402 (65.5%)	
**Age at delivery (years)**				
< 26 years	2901 (45.0%)	7957 (39.6%)	10,858 (40.9%)	< 0.001***
≥ 26 years	3542 (55.0%)	12,151 (60.4%)	15,693 (59.1%)	
**Race/ethnicity**				
White	4889 (75.9%)	14,763 (73.4%)	19,652 (74.0%)	< 0.001***
Non-white/Unknown	1554 (24.1%)	5345 (26.6%)	6899 (26.0%)	
**OBCMI Score**				
OBCMI score ≤ 2	5084 (78.9%)	14,416 (71.7%)	19,500 (73.4%)	< 0.001***
OBCMI score > 2	1359 (21.1%)	5692 (28.3%)	7051 (26.6%)	

^a^Boldface indicates statistical significance (*p < 0.05, **p < 0.001, ***p < 0.001)

### Treatment rates: enrollees with PMAD diagnoses only

**Fig. 2:**
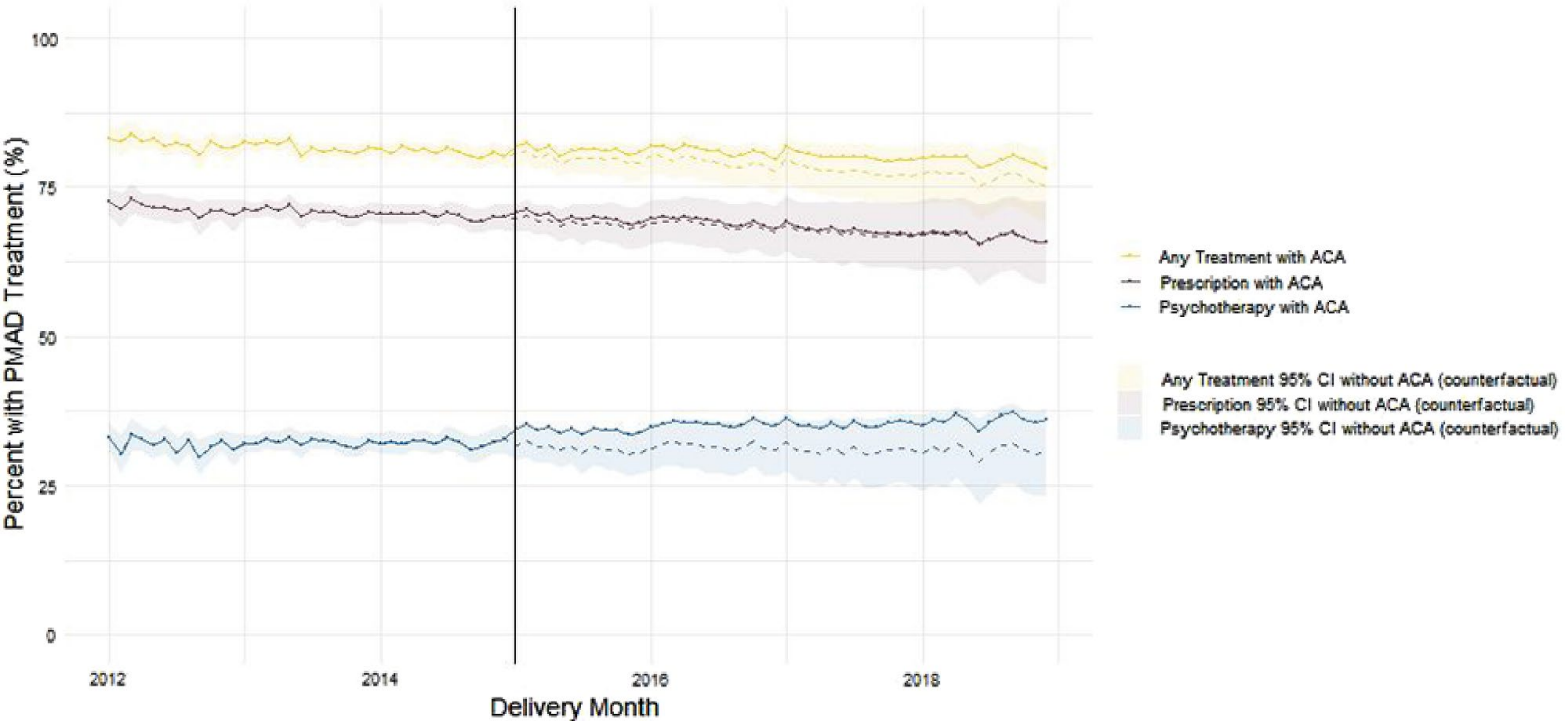
Monthly treatment rates among birthing enrollees with perinatal mood and anxiety disorder (PMAD) diagnosis 2012–2018^a,b^. ^a^Adjusted for: time, ACA implementation, time since ACA implementation, percent of enrollees > 26, percent of non-white enrollees, and percent of enrollees with OBCMI score > 2. ^b^The shading and dashed lines indicate model with ACA implementation set to 0; this represents a counterfactual projection of rates had the ACA not been implemented

Figure 2 displays adjusted monthly PMAD treatment rates by treatment modality among participants with PMAD diagnoses. These rates adjust for time, ACA implementation, and time since ACA implementation as well as enrollee demographics and indicate the percent of enrollees with a PMAD diagnosis who received a PMAD treatment at any point during the perinatal period plotted by infant birth month.

Pre-ACA adjusted treatment rates decreased by 0.12% points per month (95% CI: -0.22 to -0.02; *p* = 0.022) for the “any treatment” category (i.e., either psychotherapy or prescription medication); however, the Pre-ACA adjusted treatment rates were statistically stable when psychotherapy and prescription medication were modeled separately. Time and Time Since ACA did not have an association with any of these treatment types, indicating that ACA is not associated with changes in any treatment type. The unadjusted and adjusted models appear in Appendix F.

In the main analysis, we included only one birth per participant by randomly selecting just one birth for enrollees with more than one birth during the study period. Sensitivity analyses on all births (including multiple births to the same individual) and sensitivity analyses on only the first birth to appear in the dataset each yielded similar results.

## Conclusions

ACA implementation was associated with increased PMAD diagnosis rates among birthing Michigan Medicaid enrollees; however, ACA implementation was not associated with changes in treatment rates among Michigan Medicaid enrollees with a PMAD diagnosis. These results indicate that policy changes and clinical care enhancements related to ACA implementation had a greater impact on detection, diagnosis, and documentation than treatment utilization or access.

### Prevalence versus detection

These results indicate that PMAD diagnosis rates increased by almost a percentage point after ACA implementation. However, whether this reflects increasing prevalence, improved detection, or a combination of both remains difficult to parse. Several studies indicate that the ACA decreased depressive symptoms in the general and birthing population [17, 25, 26, 32]. However, the ACA also expanded coverage for mental health screening and increased access to prenatal care visits (which typically include mental health screening) [6, 22–24]. Historically, PMADs remain underdiagnosed [11], so it’s conceivable that even if depressive symptoms became less prevalent, improved detection would result in the increased diagnosis rates observed in this study.

### Long-term trends

Diagnosis rates demonstrated significant and increasing month-to-month temporal trends in both the Pre-ACA and Post-ACA time periods. However, Post-ACA diagnosis rates increased at a slower rate than Pre-ACA diagnosis rates in both adjusted and unadjusted analyses.

Even with perfect detection, the increasing diagnosis rates observed in the Pre-ACA period would not have continued in perpetuity (since PMADs do not afflict the entire population). Literature estimates PMADs affect roughly one in five births [33–36], and our models indicate a Post-ACA diagnosis rate of 16.8% of all births. As the clinical diagnosis rate approaches the true rate of disease, a natural slowing of diagnosis may occur, reflecting a diagnosis “ceiling.”

As diagnosis rates increase, the risk profile, relevant barriers, and characteristics of undiagnosed cases may change, as these reflect the most difficult-to-detect cases. Stakeholders should continue assessing facilitators, barriers, and factors associated with underdiagnosis as the healthcare landscape evolves.

### Stagnant PMAD treatment rates

Treatment rates among enrollees diagnosed with PMADs remained relatively consistent at roughly a 30% psychotherapy treatment rate and 70% prescription medication treatment rate. Other studies indicate that although adults with depression had a decreased likelihood of delaying mental health services due to cost after ACA implementation [32], they did not have an increased likelihood of using mental health services after ACA implementation [10]. Han, Lai, and Yu found that Medicaid expansion may have increased the *amount* of care used by mental health service users but not the *number* of mental health service users [37].

The results of this study did not evaluate the nuanced difference between amount of mental health services per user versus number of users of mental health service, nor did this study evaluate whether care was guideline concordant. Rather, this analysis used a broad definition which only identified the number of users of mental health service. Future studies should determine whether the ACA impacted amount and adequacy of treatment among enrollees diagnosed with PMADs.

The non-significant effect of ACA on treatment rates observed in this study may indicate that the ACA did not eliminate the most pervasive barriers to treatment. Although lack of health insurance represents a major barrier to care, other factors not addressed by the ACA remain. While the ACA has effectively reduced many barriers to care, barriers that persist include lack of time, transportation, or childcare; stigma/patient treatment preference; and poor patient/provider relationships [12–15].

The literature indicates that routine care and perinatal mental health screening serve as an effective strategy to identify individuals with PMAD, but treating individuals with PMAD requires more intensive intervention [38–40]. Structural improvements like increasing number of mental health providers, integrating mental health providers into obstetric clinics, and disseminating culturally appropriate training may have a greater likelihood of increasing treatment. Although the ACA supports such initiatives with expanded funding and incentives, these changes take longer to implement, and observable impacts would occur over a much longer timeframe than observed in this study.

### Strengths

A major strength of this study includes the use of a large, comprehensive cohort of all birthing Michigan Medicaid enrollees. Having access to an entire study population maximizes generalizability and minimizes selection biases. ITS analysis remains the ideal approach for studying macro-level changes in which multiple aspects of healthcare change simultaneously, as in this case where specific aspects of ACA policies cannot be studied in isolation. The extended timeframe of these data allows for a relatively large number of time points both before and after intervention.

### Limitations

Two primary weaknesses of the ITS analytic approach are the lack of a contemporaneous control group and the inability to control for other contemporaneous changes unrelated to the intervention of interest. For example, factors like the October 2015 transition from ICD 9 to ICD 10 [41] or the May 2015 American College of Obstetrics and Gynecology (ACOG) recommendation to conduct universal perinatal mental health screening [42] may have affected the observed pre-to-post effects. The continuous enrollment study criterion ensures an equal observation of all participants but excludes enrollees with discontinuity of care and renders results less generalizable. This criterion may disproportionality impact the Pre-ACA period, when enrollees had a higher susceptibility to insurance churn. Finally, this study looks specifically at birthing people enrolled in Medicaid, a population which differs from other populations (i.e., privately insured, uninsured). Due to the limited observation window, we could not assess important factors which may predate pregnancy, such as gravidity, parity, or mental health history, both of which may have impacted likelihood of diagnosis or treatment.

## Conclusion

The ACA represents the largest health care policy initiative in decades and aimed to expand coverage, improve clinical care, and enhance health care infrastructure. PMADs are frequently underdiagnosed. PMAD diagnosis rates increased in Michigan Medicaid enrollees after ACA implementation, possibly indicating an improved rate of detection. However, PMAD treatment rates remained unchanged for those with a diagnosed PMAD, and psychotherapy continues to exhibit minimal reach compared to prescription medications. Policy initiatives associated with ACA implementation likely improved clinical documentation, but additional supports are necessary to mitigate treatment barriers.

### Electronic supplementary material

Below is the link to the electronic supplementary material.

**Supplementary Material 1:** Appendices

## Data Availability

The data that support the findings of this study are available from the Michigan Department of Health and Human Services, but restrictions apply to the availability of these data, which were used under license for the current study, and so are not publicly available. Data are however available from the authors (corresponding author: Stephanie V. Hall, stephall@med.umich.edu) upon reasonable request and with permission of the Michigan Department of Health and Human Services.

## Abbreviations

ACA: Patient Protection and Affordable Care Act
ACOG: American College of Obstetrics and Gynecology
CPT: Current Procedural Terminology
FPL: Federal poverty level
HCUP: Healthcare Utilization Project
HEDIS: Healthcare Effectiveness Data and Information Set
HIC: Hierarchical Ingredient Code
ICD: International Classification of Diseases
MDHHS: Michigan Department of Health and Human Services
OBCMI: Obstetric Comorbidity Index
PMAD: Perinatal mood and anxiety disorder
SMM: Severe maternal morbidity
US: United States
