# Supplementary material for: The impact of the affordable care act on perinatal mood and anxiety disorder diagnosis and treatment rates among Michigan Medicaid enrollees 2012–2018
Source: BMC Health Serv Res. 2024 Jan 30;24:149. doi: 10.1186/s12913-023-10539-y (PMC10826065; doi:10.1186/s12913-023-10539-y)
Supplement: Supplementary file 1 — Supplementary Material 1: Appendices [file 12913_2023_10539_MOESM1_ESM.docx]

# List of Supplemental Appendices

###### Appendix A: List of International Classification of Disease (ICD)-9 and ICD-10 Codes Used to Identify Mood and Anxiety Disorder Diagnoses

###### Appendix B: List of Hierarchical Ingredient Code (HIC) Numbers Used to Identify Antidepressant, Anxiolytic, and Mood Stabilizing Prescription Claims

###### Appendix C: List of Current Procedural Terminology (CPT) Codes Used to Identify Psychotherapy Claims

###### Appendix D: Study Cohort Diagram

###### Appendix E: Linear Regression Interrupted Time Series (ITS) Analysis of Monthly Diagnosis Rates (n=84 months)

###### Appendix F: Linear Regression Interrupted Time Series (ITS) Analysis of Monthly Treatment Rates Among Only Those with Perinatal Mood and/or Anxiety Disorder Diagnosis (n=84 months)

###### Appendix A: List of International Classification of Disease (ICD)-9 and ICD-10 Codes Used to Identify Mood and Anxiety Disorder Diagnoses

| Code Type | Code | Code Description |
| --- | --- | --- |
| ICD-9-CM | 3003 | Obsessive-compulsive disorders |
| ICD-9-CM | 3005 | Neurasthenia |
| ICD-9-CM | 3009 | Unspecified nonpsychotic mental disorder |
| ICD-9-CM | 3080 | Predominant disturbance of emotions |
| ICD-9-CM | 3081 | Predominant disturbance of consciousness |
| ICD-9-CM | 3082 | Predominant psychomotor disturbance |
| ICD-9-CM | 3083 | Other acute reactions to stress |
| ICD-9-CM | 3084 | Mixed disorders as reaction to stress |
| ICD-9-CM | 3089 | Unspecified acute reaction to stress |
| ICD-9-CM | 3130 | Overanxious disorder specific to childhood and adolescence |
| ICD-9-CM | 3131 | Misery and unhappiness disorder specific to childhood and adolescence |
| ICD-9-CM | 3133 | Relationship problems specific to childhood and adolescence |
| ICD-9-CM | 29384 | Anxiety disorder in conditions classified elsewhere |
| ICD-9-CM | 30000 | Anxiety state, unspecified |
| ICD-9-CM | 30001 | Panic disorder without agoraphobia |
| ICD-9-CM | 30002 | Generalized anxiety disorder |
| ICD-9-CM | 30009 | Other anxiety states |
| ICD-9-CM | 30010 | Hysteria, unspecified |
| ICD-9-CM | 30020 | Phobia, unspecified |
| ICD-9-CM | 30021 | Agoraphobia with panic disorder |
| ICD-9-CM | 30022 | Agoraphobia without mention of panic attacks |
| ICD-9-CM | 30023 | Social phobia |
| ICD-9-CM | 30029 | Other isolated or specific phobias |
| ICD-9-CM | 30089 | Other somatoform disorders |
| ICD-9-CM | 31321 | Shyness disorder of childhood |
| ICD-9-CM | 31322 | Introverted disorder of childhood |
| ICD-9-CM | 31382 | Identity disorder of childhood or adolescence |
| ICD-9-CM | 31383 | Academic underachievement disorder of childhood or adolescence |
| ICD-10-CM | F064 | Anxiety disorder due to known physiological condition |
| ICD-10-CM | F4000 | Agoraphobia, unspecified |
| ICD-10-CM | F4001 | Agoraphobia with panic disorder |
| ICD-10-CM | F4002 | Agoraphobia without panic disorder |
| ICD-10-CM | F4010 | Social phobia, unspecified |
| ICD-10-CM | F4011 | Social phobia, generalized |
| ICD-10-CM | F40210 | Arachnophobia |
| ICD-10-CM | F40218 | Other animal type phobia |
| ICD-10-CM | F40220 | Fear of thunderstorms |
| ICD-10-CM | F40228 | Other natural environment type phobia |
| ICD-10-CM | F40230 | Fear of blood |
| ICD-10-CM | F40231 | Fear of injections and transfusions |
| ICD-10-CM | F40232 | Fear of other medical care |
| ICD-10-CM | F40233 | Fear of injury |
| ICD-10-CM | F40240 | Claustrophobia |
| ICD-10-CM | F40241 | Acrophobia |
| ICD-10-CM | F40242 | Fear of bridges |
| ICD-10-CM | F40243 | Fear of flying |
| ICD-10-CM | F40248 | Other situational type phobia |
| ICD-10-CM | F40290 | Androphobia |
| ICD-10-CM | F40291 | Gynephobia |
| ICD-10-CM | F40298 | Other specified phobia |
| ICD-10-CM | F408 | Other phobic anxiety disorders |
| ICD-10-CM | F409 | Phobic anxiety disorder, unspecified |
| ICD-10-CM | F410 | Panic disorder [episodic paroxysmal anxiety] |
| ICD-10-CM | F411 | Generalized anxiety disorder |
| ICD-10-CM | F413 | Other mixed anxiety disorders |
| ICD-10-CM | F418 | Other specified anxiety disorders |
| ICD-10-CM | F419 | Anxiety disorder, unspecified |
| ICD-10-CM | F42 | Obsessive-compulsive disorder |
| ICD-10-CM | F422 | Mixed obsessional thoughts and acts |
| ICD-10-CM | F423 | Hoarding disorder |
| ICD-10-CM | F424 | Excoriation (skin-picking) disorder |
| ICD-10-CM | F428 | Other obsessive-compulsive disorder |
| ICD-10-CM | F429 | Obsessive-compulsive disorder, unspecified |
| ICD-10-CM | F430 | Acute stress reaction |
| ICD-10-CM | F488 | Other specified nonpsychotic mental disorders |
| ICD-10-CM | F489 | Nonpsychotic mental disorder, unspecified |
| ICD-10-CM | R452 | Unhappiness |
| ICD-10-CM | R453 | Demoralization and apathy |
| ICD-10-CM | R454 | Irritability and anger |
| ICD-10-CM | R455 | Hostility |
| ICD-10-CM | R456 | Violent behavior |
| ICD-10-CM | R457 | State of emotional shock and stress, unspecified |
| ICD-10-CM | R4581 | Low self-esteem |
| ICD-10-CM | R4582 | Worries |
| ICD-10-CM | R4583 | Excessive crying of child, adolescent or adult |
| ICD-10-CM | R4584 | Anhedonia |
| ICD-9-CM | 311 | Depressive disorder, not elsewhere classified |
| ICD-9-CM | 3004 | Dysthymic disorder |
| ICD-9-CM | 2962 | Major depressive affective disorder, single episode, in partial or unspecified remission |
| ICD-9-CM | 29620 | Major depressive affective disorder, single episode, in partial or unspecified remission |
| ICD-9-CM | 29621 | Major depressive affective disorder, single episode, in partial or unspecified remission |
| ICD-9-CM | 29622 | Major depressive affective disorder, single episode, in partial or unspecified remission |
| ICD-9-CM | 29623 | Major depressive affective disorder, single episode, in partial or unspecified remission |
| ICD-9-CM | 29624 | Major depressive affective disorder, single episode, in partial or unspecified remission |
| ICD-9-CM | 29625 | Major depressive affective disorder, single episode, in partial or unspecified remission |
| ICD-9-CM | 29626 | Major depressive affective disorder, single episode, in full remission |
| ICD-9-CM | 29630 | Major depressive affective disorder, recurrent episode, unspecified |
| ICD-9-CM | 29631 | Major depressive affective disorder, recurrent episode, mild |
| ICD-9-CM | 29632 | Major depressive affective disorder, recurrent episode, moderate |
| ICD-9-CM | 29633 | Major depressive affective disorder, recurrent episode, severe, without mention of psychotic behavior |
| ICD-9-CM | 29634 | Major depressive affective disorder, recurrent episode, severe, specified as with psychotic behavior |
| ICD-9-CM | 29635 | Major depressive affective disorder, recurrent episode, in partial or unspecified remission |
| ICD-9-CM | 29636 | Major depressive affective disorder, recurrent episode, in full remission |
| ICD-9-CM | 29682 | Atypical depressive disorder |
| ICD-10-CM | F320 | Major depressive disorder, single episode, mild |
| ICD-10-CM | F321 | Major depressive disorder, single episode, moderate |
| ICD-10-CM | F322 | Major depressv disord, single epsd, sev w/o psych features |
| ICD-10-CM | F323 | Major depressv disord, single epsd, severe w psych features |
| ICD-10-CM | F324 | Major depressv disorder, single episode, in partial remis |
| ICD-10-CM | F325 | Major depressive disorder, single episode, in full remission |
| ICD-10-CM | F328 | Other depressive episodes |
| ICD-10-CM | F3289 | Other specified depressive episodes |
| ICD-10-CM | F329 | Major depressive disorder, single episode, unspecified |
| ICD-10-CM | F330 | Major depressive disorder, recurrent, mild |
| ICD-10-CM | F331 | Major depressive disorder, recurrent, moderate |
| ICD-10-CM | F332 | Major depressv disorder, recurrent severe w/o psych features |
| ICD-10-CM | F333 | Major depressv disorder, recurrent, severe w psych symptoms |
| ICD-10-CM | F3340 | Major depressive disorder, recurrent, in remission, unsp |
| ICD-10-CM | F3341 | Major depressive disorder, recurrent, in partial remission |
| ICD-10-CM | F3342 | Major depressive disorder, recurrent, in full remission |
| ICD-10-CM | F338 | Other recurrent depressive disorders |
| ICD-10-CM | F339 | Major depressive disorder, recurrent, unspecified |

###### Appendix B: List of Hierarchical Ingredient Code (HIC) Numbers Used to Identify Antidepressant, Anxiolytic, and Mood Stabilizing Prescription Claims

| Code | Code Category | Code Description |
| --- | --- | --- |
| H24 | Antidepressant | ANTIDEPRESSANT - POSTPARTUM DEPRESSION (PPD) |
| H2H | Antidepressant | MONOAMINE OXIDASE (MAO) INHIBITOR ANTIDEPRESSANTS |
| H2H | Antidepressant | MONOAMINE OXIDASE (MAO) INHIBITORS |
| H2H | Antidepressant | MONOAMINE OXIDASE(MAO) INHIBITORS |
| H2J | Antidepressant | ANTIDEPRESSANTS O.U. |
| H2J | Antidepressant | ANTIDEPRESSANTS |
| H2K | Antidepressant | ANTIDEPRESSANT COMBINATIONS |
| H2K | Antidepressant | ANTIDEPRESSANT COMBINATIONS O.U. |
| H2N | Antidepressant | ANTIDEPRESSANTS (CONTINUED 1) |
| H2N | Antidepressant | ANTIDEPRESSANTS O.U. (CONTINUED 1) |
| H2S | Antidepressant | SELECTIVE SEROTONIN REUPTAKE INHIBITOR (SSRIS) |
| H2U | Antidepressant | TRICYCLIC ANTIDEPRESSANTS,REL.NON-SEL.REUPT-INHIB |
| H2U | Antidepressant | TRICYCLIC ANTIDEPRESSANTS & REL. NON-SEL. RU-INHIB |
| H2Y | Antidepressant | TRICYCLIC ANTIDEPRESSANT-NON-PHENOTHIAZINE COMB. |
| H2Y | Antidepressant | TRICYCLIC ANTIDEPRESSANT/NON-PHENOTHIAZINE COMB. |
| H7B | Antidepressant | ALPHA-2 RECEPTOR ANTAGONIST ANTIDEPRESSANTS |
| H7C | Antidepressant | SEROTONIN-NOREPINEPHRINE REUPTAKE-INHIB (SNRIS) |
| H7D | Antidepressant | NOREPINEPHRINE AND DOPAMINE REUPTAKE INHIB (NDRIS) |
| H7E | Antidepressant | SEROTONIN-2 ANTAGONIST/REUPTAKE INHIBITORS (SARIS) |
| H7I | Antidepressant | ANTIDEPRESSANT O.U./BARB/BELLADONNA ALKALOID COMB |
| H7I | Antidepressant | ANTIDEPRESSANT/BARB/BELLADONNA ALKALOID COMB |
| H7J | Antidepressant | MAOIS -NON-SELECTIVE,IRREVERSIBLE ANTIDEPRESSANTS |
| H7J | Antidepressant | MAOIS - NON-SELECTIVE & IRREVERSIBLE |
| H7J | Antidepressant | MAOIS - NON-SELECTIVE AND IRREVERSIBLE |
| H7K | Antidepressant | MAOIS - A SELECTIVE AND REVERSIBLE (RIMA) |
| H7K | Antidepressant | MAOIS - A SELECTIVE & REVERSIBLE (RIMA) |
| H7L | Antidepressant | MAOI N-S & IRREVERSIBLE/PHENOTHIAZINE COMBINATNS |
| H7L | Antidepressant | MAOI NON-SELECTIVE,IRREVERSIBLE-PHENOTHIAZINE COMB |
| H8P | Antidepressant | SSRI AND 5HT1A PARTIAL AGONIST ANTIDEPRESSANTS |
| H8P | Antidepressant | SSRI & 5HT1A PARTIAL AGONIST ANTIDEPRESSANT |
| H8S | Antidepressant | ANTIDEPRESSANTS O.U. (CONTINUED 2) |
| H8S | Antidepressant | ANTIDEPRESSANTS (CONTINUED 2) |
| H8T | Antidepressant | SSRI, SEROTONIN RECEPTOR MODULATOR ANTIDEPRESSANTS |
| H8T | Antidepressant | SSRI & SEROTONIN RECEPTOR MODULATOR ANTIDEPRESSANT |
| H8Z | Antidepressant | ANTIDEPRESSANT - NMDA RECEPTOR ANTAGONIST |
| H2W | Antidepressant/antipsychotic | TRICYCLIC ANTIDEPRESSANT-PHENOTHIAZINE COMBINATNS |
| H2W | Antidepressant/antipsychotic | TRICYCLIC ANTIDEPRESSANT/PHENOTHIAZINE COMBINATNS |
| H7A | Antidepressant/antipsychotic | TRICYCLIC ADP/PHENOTHIAZINE/BENZODIAZEPINE COMB. |
| H2X | Antidepressant/anxiolytic | TRICYCLIC ANTIDEPRESSANT-BENZODIAZEPINE COMBINATNS |
| H2X | Antidepressant/anxiolytic | TRICYCLIC ANTIDEPRESSANT/BENZODIAZEPINE COMBINATNS |
| H7M | Antidepressant/anxiolytic | ANTIDEPRESSANT O.U./CARBAMATE ANXIOLYTIC COMBINATN |
| H7M | Antidepressant/anxiolytic | ANTIDEPRESSANT/CARBAMATE ANXIOLYTIC COMBINATION |
| H20 | Anxiolytic | ANTI-ANXIETY - BENZODIAZEPINES |
| H2F | Anxiolytic | ANTI-ANXIETY DRUGS |
| H2P | Anxiolytic | ANTI-ANXIETY DRUGS (CONTINUED 1) |
| H8A | Anxiolytic | ANTI-ANXIETY (ANXIOLYTIC) AND ANTISPASMODIC COMB. |
| H8A | Anxiolytic | ANTI-ANXIETY(BENZODIAZEPINE)AND ANTISPASMODIC COMB |
| H8K | Anxiolytic | ANTI-ANXIETY DRUGS/DIETARY SUPPLEMENT COMBINATIONS |
| H2M | Mood stabilizer | BIPOLAR DISORDER DRUGS |
| H2M | Mood stabilizer | ANTI-MANIA DRUGS |

###### Appendix C: List of Current Procedural Terminology (CPT) Codes Used to Identify Psychotherapy Claims

| Code Type | Code | Code Description |
| --- | --- | --- |
| CPT/HPCS | 90804 | PSYTX, OFFICE, 20-30 MIN |
| CPT/HPCS | 90805 | PSYTX, OFF, 20-30 MIN W/E&M |
| CPT/HPCS | 90806 | PSYTX, OFF, 45-50 MIN |
| CPT/HPCS | 90807 | PSYTX, OFF, 45-50 MIN W/E&M |
| CPT/HPCS | 90808 | PSYTX, OFFICE, 75-80 MIN |
| CPT/HPCS | 90809 | PSYTX, OFF, 75-80, W/E&M |
| CPT/HPCS | 90810 | INTAC PSYTX, OFF, 20-30 MIN |
| CPT/HPCS | 90811 | INTAC PSYTX, 20-30, W/E&M |
| CPT/HPCS | 90812 | INTAC PSYTX, OFF, 45-50 MIN |
| CPT/HPCS | 90813 | INTAC PSYTX, 45-50 MIN W/E&M |
| CPT/HPCS | 90814 | INTAC PSYTX, OFF, 75-80 MIN |
| CPT/HPCS | 90815 | INTAC PSYTX, 75-80 W/E&M |
| CPT/HPCS | 90816 | PSYTX, HOSP, 20-30 MIN |
| CPT/HPCS | 90817 | PSYTX, HOSP, 20-30 MIN W/E&M |
| CPT/HPCS | 90818 | PSYTX, HOSP, 45-50 MIN |
| CPT/HPCS | 90819 | PSYTX, HOSP, 45-50 MIN W/E&M |
| CPT/HPCS | 90820 | DIAGNOSTIC INTERVIEW |
| CPT/HPCS | 90821 | PSYTX, HOSP, 75-80 MIN |
| CPT/HPCS | 90822 | PSYTX, HOSP, 75-80 MIN W/E&M |
| CPT/HPCS | 90823 | INTAC PSYTX, HOSP, 20-30 MIN |
| CPT/HPCS | 90824 | INTAC PSYTX, HSP 20-30 W/E&M |
| CPT/HPCS | 90826 | INTAC PSYTX, HOSP, 45-50 MIN |
| CPT/HPCS | 90827 | INTAC PSYTX, HSP 45-50 W/E&M |
| CPT/HPCS | 90828 | INTAC PSYTX, HOSP, 75-80 MIN |
| CPT/HPCS | 90829 | INTAC PSYTX, HSP 75-80 W/E&M |
| CPT/HPCS | 90832 | PSYTX W PT 30 MINUTES |
| CPT/HPCS | 90833 | PSYTX W PT W E/M 30 MIN |
| CPT/HPCS | 90834 | PSYTX W PT 45 MINUTES |
| CPT/HPCS | 90836 | PSYTX W PT W E/M 45 MIN |
| CPT/HPCS | 90837 | PSYTX W PT 60 MINUTES |
| CPT/HPCS | 90838 | PSYTX W PT W E/M 60 MIN |
| CPT/HPCS | 90839 | PSYTX CRISIS INITIAL 60 MIN |
| CPT/HPCS | 90840 | PSYTX CRISIS EA ADDL 30 MIN |
| CPT/HPCS | 90841 | PSYCHOTHERAPY |
| CPT/HPCS | 90842 | PSYCHOTHERAPY 75-80 MIN |
| CPT/HPCS | 90843 | PSYCHOTHERAPY 20-30 MIN. |
| CPT/HPCS | 90844 | PSYCHOTHERAPY 45-50 MIN. |
| CPT/HPCS | 90846 | FAMILY PSYTX W/O PT 50 MIN |
| CPT/HPCS | 90847 | FAMILY PSYTX W/PT 50 MIN |
| CPT/HPCS | 90849 | MULTIPLE FAMILY GROUP PSYTX |
| CPT/HPCS | 90853 | GROUP PSYCHOTHERAPY |
| CPT/HPCS | 90855 | INDIVIDUAL PSYCHOTHERAPY |


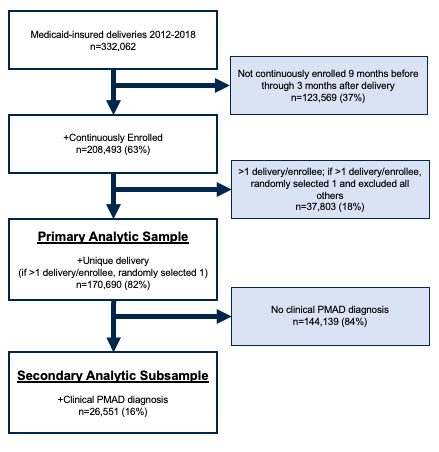


###### Appendix D: Study Cohort Diagram

###### Appendix E: Linear Regression Interrupted Time Series (ITS) Analysis of Monthly Diagnosis Rates (n=84 months)

|  | **UNADJUSTED ITS OF**  **MONTHLY DIAGNOSIS RATES** | **ADJUSTED ITS OF**  **MONTHLY DIAGNOSIS RATES** |
| --- | --- | --- |
|  | **Odds Ratio** | **Odds Ratio** |
|  | **(95% CI)** | **(95% CI)** |
| Intercept | **10.28***** | **10.80***** |
|  | **(9.76, 10.80)** | **(5.11, 16.49)** |
| Time | **0.11***** | **0.08***** |
|  | **(0.08, 0.13)** | **(0.05, 0.12)** |
| ACA Implementation | **1.01**** | **0.76*** |
|  | **(0.33, 1.68)** | **(0.01, 1.52)** |
| Time Since ACA Implementation | **-0.04**** | **-0.04**** |
|  | **(-0.07, -0.02)** | **(-0.07, -0.01)** |
| Percent Enrollees >26 | . | -0.05 |
|  | . | (-0.12, 0.03) |
| Percent of Non-White Enrollees | . | -0.01 |
|  | . | (-0.13, 0.10) |
| Percent Enrollees OBCMI >2 | . | **0.25*** |
|  | . | **(0.06, 0.45)** |
| N | 84 | 84 |
| R2 | 0.914 | 0.921 |

*** p<0.001

** p<0.01

* p<0.05

###### Appendix F: Linear Regression Interrupted Time Series (ITS) Analysis of Monthly Treatment Rates Among Only Those with Perinatal Mood and/or Anxiety Disorder Diagnosis (n=84 months)

|  | **UNADJUSTED**  **ITS OF MONTHLY TREATMENT RATES** | | | **ADJUSTED**  **ITS OF MONTHLY TREATMENT RATES** | | |
| --- | --- | --- | --- | --- | --- | --- |
|  | **Psychotherapy** | **Prescription Medication** | **Any Treatment** | **Psychotherapy** | **Prescription Medication** | **Any Treatment** |
|  | **Odds Ratio**  **(95% CI)** | **Odds Ratio**  **(95% CI)** | **Odds Ratio**  **(95% CI)** | **Odds Ratio**  **(95% CI)** | **Odds Ratio**  **(95% CI)** | **Odds Ratio**  **(95% CI)** |
| Intercept | **31.99***** | **71.87***** | **82.88***** | **35.83***** | **70.39***** | **76.63***** |
|  | **(29.76, 34.21)** | **(69.81, 73.93)** | **(81.08, 84.69)** | **(22.88, 48.78)** | **(58.26, 82.52)** | **(66.14, 87.13)** |
| Time | 0.01 | -0.06 | -0.07 | -0.03 | -0.09 | **-0.12*** |
|  | (-0.10, 0.11) | (-0.15, 0.04) | (-0.16, 0.02) | (-0.15, 0.10) | (-0.20, 0.03) | **(-0.22, -0.02)** |
| ACA Implementation | 2.02 | 0.9 | 1.52 | 2.63 | 0.94 | 1.12 |
|  | (-0.84, 4.89) | (-1.76, 3.56) | (-0.81, 3.85) | (-0.47, 5.73) | (-1.97, 3.84) | (-1.39, 3.64) |
| Time Since ACA Implementation | 0.03 | -0.04 | 0.01 | 0.06 | -0.02 | 0.04 |
|  | (-0.09, 0.16) | (-0.16, 0.08) | (-0.09, 0.11) | (-0.07, 0.18) | (-0.14, 0.10) | (-0.06, 0.14) |
| Percent Enrollees >26 | . | . | . | 0.04 | 0.09 | 0.12 |
|  | . | . | . | (-0.16, 0.24) | (-0.10, 0.27) | (-0.04, 0.29) |
| Percent of Non-White Enrollees | . | . | . | **-0.28*** | -0.17 | -0.11 |
|  | . | . | . | **(-0.56, -0.01)** | (-0.43, 0.09) | (-0.33, 0.12) |
| Percent Enrollees OBCMI >2 | . | . | . | 0.07 | 0.07 | 0.15 |
|  | . | . | . | (-0.21, 0.36) | (-0.19, 0.34) | (-0.08, 0.38) |
| N | 84 | 84 | 84 | 84 | 84 | 84 |
| R2 | 0.2249 | 0.1984 | 0.1167 | 0.25 | 0.2416 | 0.168 |

*** p<0.001

** p<0.01

* p<0.05
